# Supplementary material for: Clinical impact of hospital distance and center transfers on adherence and outcomes in familial adenomatous polyposis: A multicenter retrospective study in a defined region of Japan
Source: PLoS One. 2026 Feb 13;21(2):e0339401. doi: 10.1371/journal.pone.0339401 (PMC12904428; doi:10.1371/journal.pone.0339401)
Supplement: S1 Fig — (DOCX) [file pone.0339401.s001.docx]

**
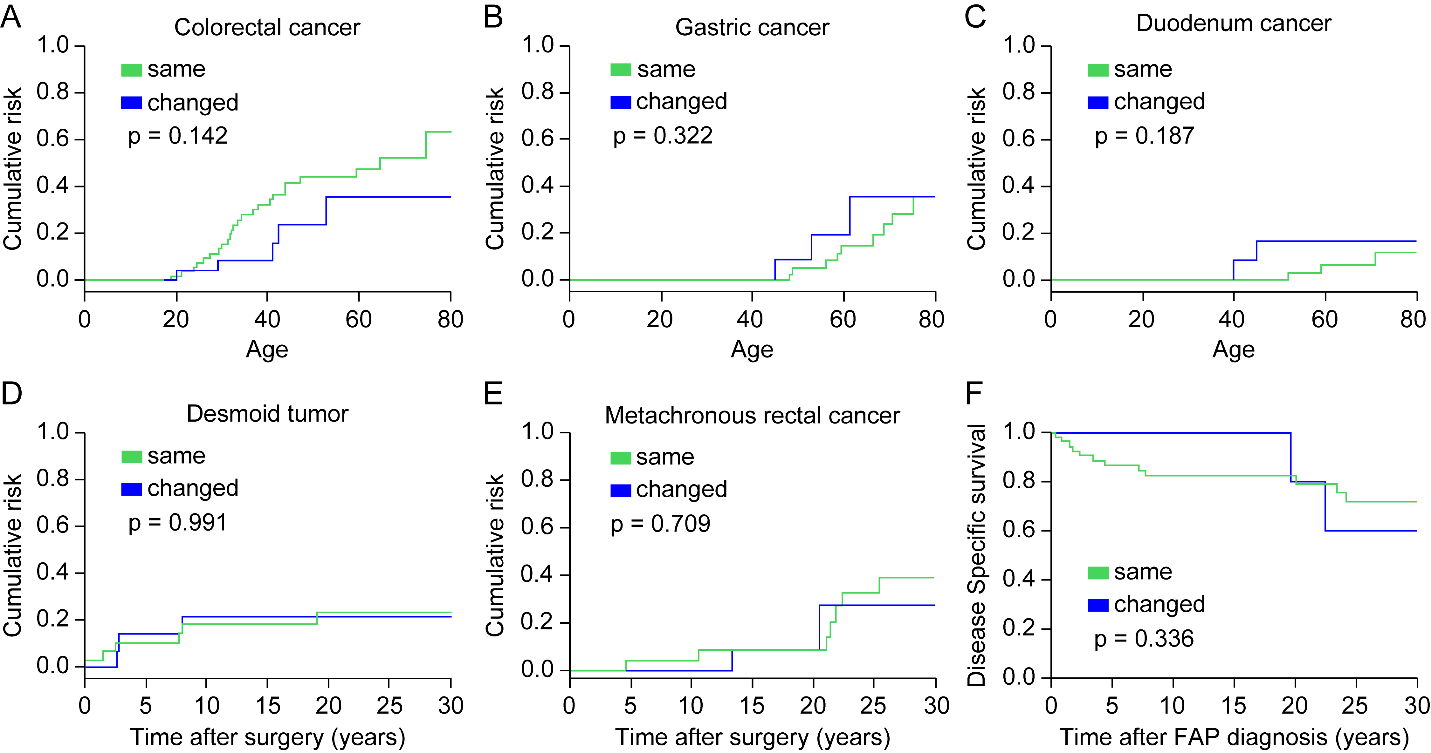
**

**S1 Figure. Comparison of cumulative risk of FAP-related tumors and survival (Gray test). (A–E)** Cumulative risk of FAP-related tumors, including (A) colorectal cancer, (B) gastric cancer, (C) duodenal cancer, (D) desmoid tumor, and (E) metachronous rectal cancer; (F) Disease-specific survival.

FAP, familial adenomatous polyposis
