# Supplementary material for: Clinical impact of hospital distance and center transfers on adherence and outcomes in familial adenomatous polyposis: A multicenter retrospective study in a defined region of Japan
Source: PLoS One. 2026 Feb 13;21(2):e0339401. doi: 10.1371/journal.pone.0339401 (PMC12904428; doi:10.1371/journal.pone.0339401)
Supplement: S1 Table — (DOCX) [file pone.0339401.s002.docx]

**S1 Table. Univariate analysis of surveillance dropout**

|  | **OR** | **95% CI** | **P-value** |
| --- | --- | --- | --- |
| **Age, ≥ 32 years** | 0.459 | 0.061–2.510 | 0.373 |
| **Sex, men** | 0.244 | 0.012–1.613 | 0.155 |
| **Family history, yes** | 0.365 | 0.063–2.121 | 0.248 |
| **Genetic test, yes** | 1.448 | 0.253–8.308 | 0.664 |
| **Initial colorectal cancer, yes** | 1.156 | 0.210–8.745 | 0.872 |
| **Surveillance hospital change, yes** | 1.043 | 0.138–5.758 | 0.963 |
| **Distant to hospital, ≥ 40 km** | 0.218 | 0.011–1.439 | 0.121 |

OR, odds ratio; CI, confidence interval.

This analysis was conducted as sensitivity analysis restricted to patients registered on or before May 31, 2022.
