# Supplementary material for: Clinical impact of hospital distance and center transfers on adherence and outcomes in familial adenomatous polyposis: A multicenter retrospective study in a defined region of Japan
Source: PLoS One. 2026 Feb 13;21(2):e0339401. doi: 10.1371/journal.pone.0339401 (PMC12904428; doi:10.1371/journal.pone.0339401)
Supplement: Attachment — Submitted filename: PLOS_Comments.docx [file pone.0339401.s005.docx]

This manuscript, *“Clinical impact of hospital distance and center transfers on adherence and outcomes in familial adenomatous polyposis: A multicenter retrospective study in a defined region of Japan,”* addresses an important and challenging topic. In a field where epidemiological studies are often hampered by the rarity of cases, the effort to integrate data across multiple centers is commendable and represents a valuable scientific contribution.

However, there are several concerns regarding the methodology and the clarity of reporting. A major concern with this design is that patients who dropped out of surveillance—likely those at the highest risk of subsequent FAP-related tumors or death—could not be observed for these outcomes. The Methods do not explain how this issue was addressed, and clarification is needed. In addition, death itself constitutes a competing risk for other outcomes; given that approximately half of the cohort had died, appropriate competing risk methods would strengthen the validity of the analyses. As this dataset is rare and valuable, I recommend consulting with a statistician to ensure that the analytic approach adequately addresses these design challenges.

The above are major concerns. I also have several minor comments regarding terminology and clarity.

Page 4 – Cohort assembly and flow
It is unclear how many cases were registered between October 1, 2024 and May 31, 2025, how many were excluded, how many underwent surgery, and how many were ultimately analyzed. It is also not specified how patients who transferred to facilities outside the study centers were handled. Please include a patient selection flow diagram detailing registration, exclusions, surgery, transfers, and final analytic cohorts.

Page 5 – Terminology (“imaging surveillance”)
“Most patients underwent annual imaging surveillance, including esophagogastroduodenoscopy, colonoscopy, and pouchoscopy” is potentially misleading, as endoscopic procedures are generally not classified as *imaging*. Please consider rephrasing as “annual endoscopic surveillance” (or “annual surveillance, including …”).

Page 5 – Terminology consistency (“withdrawal” vs “dropout”)

It is unclear whether “withdrawal from the study” and “lost to follow-up” are being used interchangeably or if they represent distinct categories (e.g., patient-initiated withdrawal vs. inability to trace the patient). In addition, the handling of deaths is not fully specified—does surveillance continuation end at the date of death?

Page 5 and 11 – Start time of surveillance
There is an inconsistency: surveillance continuation is described as beginning at diagnosis (Page 5), whereas later “surveillance” is defined as starting from surgery (Page 11, Fig. 3 legend). Please clarify these definitions, distinguish preoperative from postoperative surveillance, and ensure consistent usage across the manuscript. Since only surgical cases were included, please also explain how diagnosed but non-surgical cases were handled at the selection stage (e.g., excluded, and why).

Page 6 – Distance definition for transferred patients
For patients who changed hospitals during follow-up, please specify which distance was used in the analyses: distance to the initial hospital, the final hospital, a time-updated measure, or another definition.

Page 6 – Three-year threshold vs follow-up window
Surveillance dropout is defined as a ≥3-year absence of consultations/examinations. With follow-up ending May 31, 2025, only cases registered by May 31, 2022 can fully satisfy this definition. Please acknowledge this limitation and, if feasible, perform a sensitivity analysis (e.g., restricting to registrations ≤May 31, 2022; or using alternative thresholds) to assess robustness.

Page 7 – Comment on Surveillance Continuation Rates
It is unclear whether the reported values (86.5% at 10 years and 64.4% at 20 years) represent crude observed proportions or Kaplan–Meier estimates. Since not all patients had the opportunity to be followed for 20 years, these figures are difficult to interpret as raw proportions. It would be more appropriate to present them as Kaplan–Meier–estimated continuation rates, ideally with 95% confidence intervals.

Page 11 – Table reference mismatch
“In this database, six cases of surveillance dropouts were identified (Table 2).” However, Table 2 presents univariate logistic regression for dropout, not merely case counts. Please correct the table number (if counts are elsewhere) or revise the description to match what Table 2 actually shows.

Fig. 4A–C – Group definitions and consistent framing
The terms “changed hospital group” and “surveillance hospital changes” are ambiguous. Do they correspond to Groups II and III as defined in Fig. 4A (II = changing hospitals during surveillance; III = changing hospitals after definitive FAP diagnosis), or to another subset? Please use the Fig. 4A group framework (I–III) consistently in the text, and clearly state which groups are being compared in each analysis.

Page 12 – Causal wording
Phrases such as “impact” and “affect” imply causality. Given the observational, retrospective design, please rephrase to “association with”, “relationship to”, or “was higher/lower among …” to avoid overstating causal claims.

Page 13 – Comment on Results (Tumor Outcomes and DSS)

The paragraph describing tumor outcomes is difficult to follow because different types of analyses (distribution in Table 1 vs. cumulative risk and survival in Fig. 5) are mixed within a single sentence. I recommend restructuring this section to clearly separate:

1. comparisons of distribution/incidence between groups (Table 1),
2. cumulative risk estimates using Kaplan–Meier analysis (Fig. 5A–E), and
3. survival outcomes (DSS; Fig. 5F).

Such restructuring will improve clarity and readability.

Fig. 5 – Difficult to interpret due to inconsistencies in labeling and definitions

Fig. 5A–E: These panels are described as showing the cumulative incidence of colorectal, gastric, duodenal, metachronous rectal, and desmoid tumors, calculated from diagnosis (Page 6). However, the x-axes are variously labeled as age or years after surgery, which creates confusion about the actual time scale used.

Fig. 5E: The y-axis is labeled as overall survival (OS), but according to the text, this figure should represent the cumulative risk of metachronous rectal cancer.

These discrepancies make it difficult for readers to interpret the results correctly. The authors should ensure that each panel has a consistent and accurate definition of both the x-axis (time scale: from diagnosis vs. from surgery) and y-axis (cumulative risk vs. survival).

Page 15 (Table 4) – Methods–Results alignment
Table 4 shows important findings, but the data sources/collection methods for the variables are not described in *Methods* (e.g., medical records abstraction, registry codes, patient questionnaires). Please add the corresponding Methods description to ensure consistency and reproducibility.

Page 16 – Center characteristics
It remains unclear whether the participating hospitals are high-level centers with FAP specialists. To support interpretation, please provide basic characteristics of study hospitals (e.g., accreditation, FAP expertise, case volume, availability of specialized endoscopy/surgery).
