# Supplementary material for: Clinical impact of hospital distance and center transfers on adherence and outcomes in familial adenomatous polyposis: A multicenter retrospective study in a defined region of Japan
Source: PLoS One. 2026 Feb 13;21(2):e0339401. doi: 10.1371/journal.pone.0339401 (PMC12904428; doi:10.1371/journal.pone.0339401)
Supplement: Attachment — Submitted filename: Response to Reviewers.docx [file pone.0339401.s007.docx]

**Editorial comment**

Major comments: Have authors used AIs for drafting this manuscript? If so, it would be nice if the authors could include the statement on the use of generative AIs (which AI, in which step) in Acknowledgement section. I assume the following is likely to be AI-generated text, because these phrases seem to have lost your tone and voice: L52-56, L60-64, p16 the last two lines-p17 the first two lines.

**Response to comments:** We would like to thank Editor for your time and efforts in reviewing our manuscript and for providing comments, which have considerably helped us improve our manuscript. We have made revisions based on your comments and have provided our point-by-point responses below. We hope that our responses and revisions appropriately address your comments.

We used an AI-based tool (ChatGPT, OpenAI) exclusively for grammatical refinement and language polishing during the drafting process. All content, data interpretation, and conclusions were developed entirely by the authors. We have added the following sentence to the Acknowledgements section:

“We used ChatGPT (OpenAI) to refine the grammar and polish the language of the manuscript.” (Lines: 357-358)

Minor comments: the word “self-determined” has a different meaning. Please consider changing the word.

**Response to comments:** Thank you for your kind comment. Based on your comment, we have revised the relevant text as follows Table 4:

“Having had no issues in previous examinations, they considered further examination unnecessary, (cases, %).”

**Reviewer Comments:**

**Reviewer 1**

This manuscript, “Clinical impact of hospital distance and center transfers on adherence and outcomes in familial adenomatous polyposis: A multicenter retrospective study in a defined region of Japan,” addresses an important and challenging topic. In a field where epidemiological studies are often hampered by the rarity of cases, the effort to integrate data across multiple centers is commendable and represents a valuable scientific contribution.

However, there are several concerns regarding the methodology and the clarity of reporting. A major concern with this design is that patients who dropped out of surveillance—likely those at the highest risk of subsequent FAP-related tumors or death—could not be observed for these outcomes. The Methods do not explain how this issue was addressed, and clarification is needed. In addition, death itself constitutes a competing risk for other outcomes; given that approximately half of the cohort had died, appropriate competing risk methods would strengthen the validity of the analyses. As this dataset is rare and valuable, I recommend consulting with a statistician to ensure that the analytic approach adequately addresses these design challenges.

The above are major concerns. I also have several minor comments regarding terminology and clarity.

**Response to comments:** We would like to thank Reviewer 1 for your time and efforts in reviewing our manuscript and for providing comments, which have considerably helped us improve our manuscript. We have made revisions based on your comments and have provided our point-by-point responses below. We hope that our responses and revisions appropriately address your comments.

We have noted your comments regarding the methodological aspects and competing risk considerations. In light of your comment, the statistical analyses, including the additional competing risk analysis, were reviewed and refined under the supervision of a biostatistician (Mikihiro Shimizu), whose substantial contribution to the study led to his inclusion as a co-author.

We agree that for patients transferred to non-participating facilities, the risks of developing FAP-related tumors or death could not be accurately determined. From a statistical standpoint, further follow-up of these cases was not feasible; therefore, censoring at the date of last confirmation was deemed appropriate. We acknowledge this as a major limitation of our study.

We also agree that death itself represents a competing risk for other outcomes. Accordingly, we made the following revisions to address this point:

(1) We added a statement to the Limitations section to clarify the potential impact of unobserved outcomes (lines: 341-343).

(2) To address the issue of death as a competing risk, we performed an additional analysis using the Gray test, which accounted for death prior to the event of interest as a competing risk. This analysis yielded similar results, supporting the robustness of our findings. (Lines: 174-176, 250-251, S1 Fig)

Page 4 – Cohort assembly and flow

It is unclear how many cases were registered between October 1, 2024 and May 31, 2025, how many were excluded, how many underwent surgery, and how many were ultimately analyzed. It is also not specified how patients who transferred to facilities outside the study centers were handled. Please include a patient selection flow diagram detailing registration, exclusions, surgery, transfers, and final analytic cohorts.

**Response to comments:** Thank you for your important comments. We have revised the Methods section to clarify the registration period and patient selection process. Specifically, data collection for this study included patients diagnosed with FAP between January 1987 and September 2024 (lines: 87-79). Although data were accessed between October 1, 2024, and May 31, 2025, no additional cases registered during this period were included in the present analysis (lines: 95-96).

We have also clarified how patients who transferred to non-participating facilities were handled. These patients were censored at the time of transfer and not followed thereafter (lines: 128-129).

Furthermore, we have added a patient selection flow diagram (new Figure 1) summarizing the overall process.

New Figure 1

Page 5 – Terminology (“imaging surveillance”)

“Most patients underwent annual imaging surveillance, including esophagogastroduodenoscopy, colonoscopy, and pouchoscopy” is potentially misleading, as endoscopic procedures are generally not classified as imaging. Please consider rephrasing as “annual endoscopic surveillance” (or “annual surveillance, including …”).

**Response to comments:** Thank you for your kind comment. Per your suggestion, we have changed the description to “annual endoscopic surveillance.” (line: 116)

Page 5 – Terminology consistency (“withdrawal” vs “dropout”)

It is unclear whether “withdrawal from the study” and “lost to follow-up” are being used interchangeably or if they represent distinct categories (e.g., patient-initiated withdrawal vs. inability to trace the patient). In addition, the handling of deaths is not fully specified—does surveillance continuation end at the date of death?

**Response to comments:** Thank you for your kind comment. In this study, “withdrawal from the study” and “lost to follow-up” referred to the same condition, i.e., patients who could no longer be followed for any reason. To avoid confusion, we have unified the terminology and now use the phrase “lost to follow-up” consistently throughout the manuscript. The corresponding sentence has been revised as follows:

“Patients were monitored until death, loss to follow-up, or end of the study (May 31, 2025)” (lines: 126-127).

In this study, surveillance continuation ended at the date of death. To clarify this point, we have revised the Methods section to explicitly state this:

“Patients who died during follow-up were censored at the time of death.” (lines: 127-128).

Page 6 – Distance definition for transferred patients

For patients who changed hospitals during follow-up, please specify which distance was used in the analyses: distance to the initial hospital, the final hospital, a time-updated measure, or another definition.

**Response to comments:** Thank you for your important comments. To address your comment, we have clarified this point in the Methods section. For patients who changed hospitals during follow-up, the straight-line distances to both the initial and final hospitals were calculated and used in the respective analyses (lines: 146-148).

Page 6 – Three-year threshold vs follow-up window

Surveillance dropout is defined as a ≥3-year absence of consultations/examinations. With follow-up ending May 31, 2025, only cases registered by May 31, 2022 can fully satisfy this definition. Please acknowledge this limitation and, if feasible, perform a sensitivity analysis (e.g., restricting to registrations ≤May 31, 2022; or using alternative thresholds) to assess robustness.

**Response to comments:** Thank you for your important comments. To address the potential limitation related to the three-year definition of surveillance dropout, a sensitivity analysis was performed by restricting the cohort to patients who were registered on or before May 31, 2022 (n=77) (lines: 163-165). As a result of this analysis, similar findings were obtained, demonstrating that the study conclusions were consistent even when accounting for the follow-up window (lines: 275-276, S1 Table).

Page 7 – Comment on Surveillance Continuation Rates

It is unclear whether the reported values (86.6% at 10 years and 64.7% at 20 years) represent crude observed proportions or Kaplan–Meier estimates. Since not all patients had the opportunity to be followed for 20 years, these figures are difficult to interpret as raw proportions. It would be more appropriate to present them as Kaplan–Meier–estimated continuation rates, ideally with 95% confidence intervals.

**Response to comments:** Thank you for your important comment. As the values represent Kaplan–Meier-estimated continuation rates, we have added the corresponding 95% confidence intervals to clarify this point (lines: 183-185).

Page 11 – Table reference mismatch

“In this database, six cases of surveillance dropouts were identified (Table 2).” However, Table 2 presents univariate logistic regression for dropout, not merely case counts. Please correct the table number (if counts are elsewhere) or revise the description to match what Table 2 actually shows.

**Response to comments:** Thank you for your kind comment. The table number was mistakenly indicated as Table 2 instead of Table 1, and this has been corrected in the revised manuscript (line: 215).

Fig. 4A–C – Group definitions and consistent framing

The terms “changed hospital group” and “surveillance hospital changes” are ambiguous. Do they correspond to Groups II and III as defined in Fig. 4A (II = changing hospitals during surveillance; III = changing hospitals after definitive FAP diagnosis), or to another subset? Please use the Fig. 4A group framework (I–III) consistently in the text, and clearly state which groups are being compared in each analysis.

**Response to comments:** Thank you for your kind comments. The terms “changed hospital group” and “surveillance hospital changes” refer to Pattern II and III, respectively, as defined in the new Fig 4A. We have revised the manuscript to apply this group framework (I–III) consistently and clarified which groups are compared in each analysis (lines: 205-206)

Page 12 – Causal wording

Phrases such as “impact” and “affect” imply causality. Given the observational, retrospective design, please rephrase to “association with”, “relationship to”, or “was higher/lower among …” to avoid overstating causal claims.

**Response to comments:** Thank you for your important comments. As suggested, we have revised the causal wording throughout the manuscript (lines: 42-43, 48, 213, 228, 235, 237, 290, 298-300, 347).

Page 13 – Comment on Results (Tumor Outcomes and DSS)

The paragraph describing tumor outcomes is difficult to follow because different types of analyses (distribution in Table 1 vs. cumulative risk and survival in Fig. 5) are mixed within a single sentence. I recommend restructuring this section to clearly separate:

1.	comparisons of distribution/incidence between groups (Table 1),

2.	cumulative risk estimates using Kaplan–Meier analysis (Fig. 5A–E), and

3.	survival outcomes (DSS; Fig. 5F).

Such restructuring will improve clarity and readability.

**Response to comments:** Thank you for your important comments. We have revised the paragraph as recommended to clearly separate the analyses for improved clarity (lines: 243-250).

Fig. 5 – Difficult to interpret due to inconsistencies in labeling and definitions

Fig. 5A–E: These panels are described as showing the cumulative incidence of colorectal, gastric, duodenal, metachronous rectal, and desmoid tumors, calculated from diagnosis (Page 6). However, the x-axes are variously labeled as age or years after surgery, which creates confusion about the actual time scale used.

**Response to comments:** Thank you for your kind comments. We have revised the description in the Methods section as suggested. We added the following sentence: “The cumulative risks of colorectal, gastric, and duodenal cancers were estimated using age as the time scale, whereas those of metachronous rectal cancer and desmoid tumors were estimated using years after surgery as the time scale, to ensure consistency with their respective clinical onset timing.” (lines: 135-138).

Fig. 5E: The y-axis is labeled as overall survival (OS), but according to the text, this figure should represent the cumulative risk of metachronous rectal cancer.

**Response to comments**: Thank you for your kind comment. We apologize for the labeling error. Per your suggestion, we have corrected the figure accordingly (the new Fig. 6E, previously Fig. 5E).

These discrepancies make it difficult for readers to interpret the results correctly. The authors should ensure that each panel has a consistent and accurate definition of both the x-axis (time scale: from diagnosis vs. from surgery) and y-axis (cumulative risk vs. survival).

**Response to comments**: Thank you for your kind comments. To address this concern, we have revised the Methods section to clarify the definitions of the x- and y-axes and the corresponding time scales (See the comment just above).

Page 15 (Table 4) – Methods–Results alignment

Table 4 shows important findings, but the data sources/collection methods for the variables are not described in Methods (e.g., medical records abstraction, registry codes, patient questionnaires). Please add the corresponding Methods description to ensure consistency and reproducibility.

**Response to comments**: Thank you for your helpful comments. We have revised the Methods section as suggested to describe the data source for variables in Table 4 (lines: 161-163).

Page 16 – Center characteristics

It remains unclear whether the participating hospitals are high-level centers with FAP specialists. To support interpretation, please provide basic characteristics of study hospitals (e.g., accreditation, FAP expertise, case volume, availability of specialized endoscopy/surgery).

**Response to comments**: Thank you for your kind comments. As there is no formal accreditation system or specialist certification specifically for FAP in Japan, it is difficult to define high-level FAP centers based on such criteria. However, all participating hospitals are accredited by the Japan Surgical Society or the Japanese Society of Gastroenterology, which ensures a high standard of surgical and gastrointestinal care. To address your comment, we have added a corresponding statement in the Methods section to clarify that these hospitals meet an adequate level of expertise for FAP management (lines: 84-87).
